# Mucocutaneous relapse during late latent syphilis as initial presentation of HIV infection

**DOI:** 10.1016/j.idcr.2024.e02062

**Published:** 2024-08-17

**Authors:** Luca Pipitò, Irene Russotto, Dalila Arena, Cinzia Calà, Antonio Cascio

**Affiliations:** a Infectious and Tropical Diseases Unit, Department of Health Promotion, Mother and Child Care, Internal Medicine and Medical Specialties "G D′Alessandro," University of Palermo, Palermo, Italy; b Palermo Fast-Track City, Casa dei Diritti, Via Libertà 45, 90143 Palermo, Italy; c Microbiology and virology Unit, Department of Health Promotion, Mother and Child Care, Internal Medicine and Medical Specialties "G D′Alessandro," University of Palermo, Palermo, Italy

**Keywords:** Late latent syphilis, HIV, Relapse

## Abstract

Syphilis is a re-emerging sexually transmitted infection. According to the definition, latent syphilis is characterized by seroreactivity without clinical manifestations. Here, we reported an atypical case of syphilis in a patient with HIV naïve to the antiretroviral treatment characterized by mucocutaneous relapse that occurred in the late latent stage. The patient reported his last sexual intercourse about 18 months ago and had self-healing genital and palmoplantar lesions more than 1 year before the presentation. He denied any other types of sexual relationship. He presented with mucocutaneous scattered lesions on his face, neck, palms, soles, penis, and scrotum. He was compliant with arthralgias, myalgias, asthenia, new onset stypsis, and mild anorectal pain. Testing for Syphilis and HIV returned positive. Opportunistic infections were excluded, and antiretroviral therapy with a bictegravir-based regimen was started. Syphilis was treated successfully with three doses of 2.4 million units of benzathine penicillin.

## Highlights

● Syphilis is a re-emerging disease.
● Late latent syphilis may be rarely associated with mucocutaneous relapses.
● Syphilis may have an atypical course in PLWH.

## Introduction

Syphilis is a sexually transmitted re-emerging infection caused by *Treponema pallidum* [1], [2]. According to the clinical manifestation, the infection may be subdivided into primary, secondary, latent, and tertiary syphilis. Primary syphilis is characterized by a chancre at the point of inoculation, and it is followed by the rash of secondary syphilis weeks later (up to 6 months). After a period of latency, up to 20 % of untreated patients may develop tertiary syphilis characterized by luetic gumma, central nervous system, and cardiovascular involvement [2], [3], [4].

Latent syphilis includes early latent within 1 year from the acquisition of infection and late latent syphilis more than 1 year from the acquisition of infection. It is characterized by seroreactivity without other evidence of primary, secondary, or tertiary disease [4].

However, mucocutaneous relapses are described in early latent syphilis but are rarely reported during late latent syphilis. To the best of our knowledge, here we reported the first case of late latent syphilis with an extensive mucocutaneous relapse in a patient with HIV infection naïve to antiretroviral treatment.

## Case presentation

**Fig. 1:**
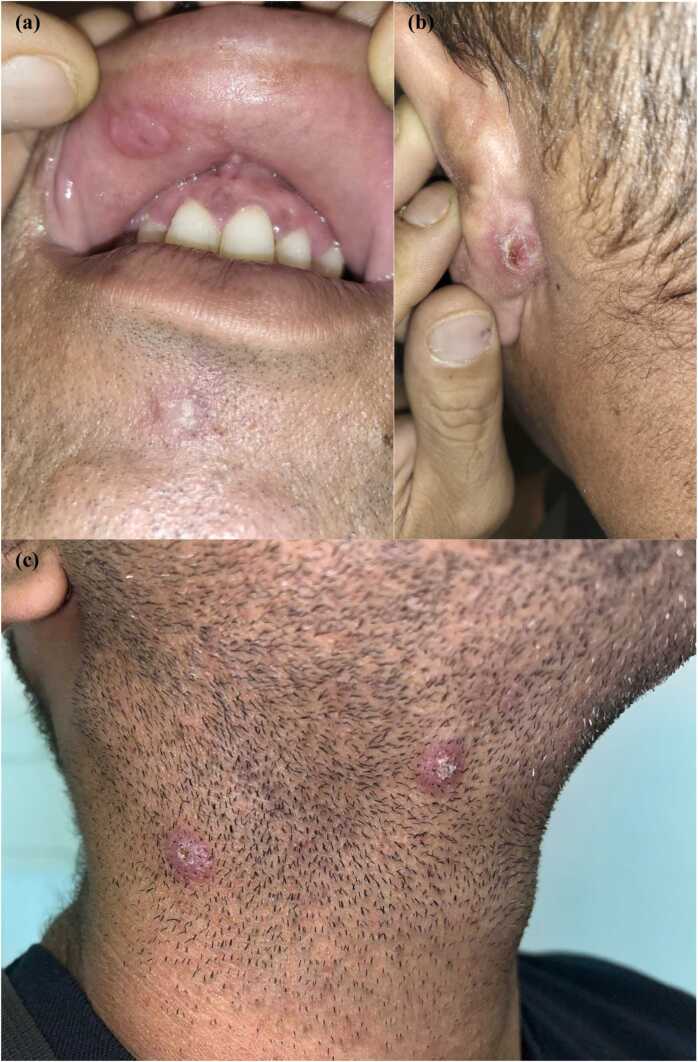
(a) Ulcerated papular lesion on the upper lip. (b) Crusted papular lesion behind the left ear. (c) Two papular lesions on the neck.

A 38-year-old Caucasian man presented a rash for 1 month that involved the face, trunk, and upper limbs. He was compliant with arthralgias, myalgias, asthenia, new onset stypsis, and mild anorectal pain. He reported his last sexual intercourse about 18 months ago, and he had self-healing genital and palmoplantar lesions more than 1 year ago. He denied any sexual relationship during the last 18 months, including oral sex and protected sexual intercourse, and he had never used illicit drugs. Physical examination showed two ulcerative lesions on the lips, painless bilateral cervical lymph nodes, and hyperkeratosis papules on the face, behind the left ear, on the neck, palms, soles, and scrotum (Fig. 1). A macular rash was in the trunk; erosive lesions were on the glans. Papular lesions were mild itching, and some of them were treated previously with cryotherapy for suspicion of common warts. He was afebrile, and his vital parameters were normal. Exams were all unremarkable but ALT (61, reference < 50 U/L). Treponemal test, rapid plasma reagin (RPR) for syphilis and serology for human immunodeficiency virus (HIV) were ordered. Testing for HIV returned positive with a CD4 count of 385 (16.96 %, CD4/ CD8 = 0.3), HIV viral load of 43,400, and negative HIV p24 antigen. Syphilis serology was positive, with an elevated RPR titer of 1:128. A diagnosis of mucocutaneous relapse during untreated late latent syphilis with HIV coinfection was made. Molecular panel for sexually transmitted infection (*Trichomonas vaginalis*, *Neisseria gonorrhoeae*, *Mycoplasma genitalium*, *Chlamydophila trachomatis*) on first-void urine and rectal swab was negative. Screening for opportunistic infections, including tuberculosis (Quantiferon Tb Gold), cryptococcosis (serum antigen), toxoplasmosis (serology), and leishmaniasis (serology and polymerase chain reaction) was unremarkable all but positive *Toxoplasma gondii* IgG serology.

The patient was treated with three doses of 2.4 million units of benzathine penicillin administered 1 per week. Antiretroviral therapy with a bictegravir-based regimen was started concurrently with the first administration. Macular rash of the trunk, glans erosions, anorectal pain, and stypsis disappeared after the first dose of penicillin. The other lesions had a progressive improvement in the next three weeks.

## Discussion

Due to the increasing incidence, syphilis is an important public health problem. Clinical features are heterogeneous, and practitioners don't always think of syphilis when new ulcers or skin lesions appear in a patient. Furthermore, there is little awareness of syphilis and its clinical manifestation among the people. In our case, the patient ignored the lesions that appeared 12 months ago, and during the relapses in the late latent stage, the lesions were wrongly treated as warts. According to the definition, late latent syphilis is not associated with clinical manifestations, although this does not mean the infection is quiescent. The Oslo study in the prepenicillin era documented that relapses occurred during latent syphilis and 90 % of them during the first 1 year. Instead, relapses were rare in late latent syphilis, and they tended to be less florid than initial manifestations [5]. Latent syphilis does not mean that infection is inactive, only that clinical signs and symptoms are not evident. The immune system may require many years before it controls the microorganism, and eradication does not happen in many instances. Stokes defined syphilis as “the relapsing disease par excellence” [4] and, although relapses occur more frequently in the early stage, we do not forget that they may also happen in the late stage with a potential for disease transmission. For example, pregnant women can transmit the infection to their infants in utero 5 or more years into the disease because episodes of silent spirochetemia may occur for prolonged periods. Castro et al. demonstrated *Treponema pallidum* DNA can be found in the blood of patients with latent syphilis [6].

According to temporal criteria, our case involved a patient with late latent syphilis, in which the immunodepression related to the mild advanced HIV infection (negative p24 antigen, CD4/CD8 ratio 0.3) contributed to the appearance of an atypical florid and scattered mucocutaneous rash as a rare relapse of syphilis during late latent stage. The patient had the last sexual intercourse 18 months before the presentation and denied any further sexual relationship. In consideration of the duration of syphilis for more than a year, we opted for 3 weekly injections of penicillin that resulted in an appropriate clinical response.

To the best of our knowledge, no other similar cases were recently reported in the literature. However, other previous reports described only ocular and bone lesions as manifestations that occurred in late latent syphilis [7], [8], [9]. Plesa et al. described a case defined as latent syphilis with a relapse characterized by hepatitis, a rash of the torso, and moth-eaten alopecia. However, the dynamic of the infection was unclear [10]. The patient recalled a previous unspecified treatment for syphilis, he was reluctant to offer a detailed sexual history, and syphilis reinfection could justify the clinical picture rather than a relapse during the late stage.

In conclusion, our case warns us to suspect syphilis in front of any rash occurring in sexually active people, and it reminds us of relapses and transmission of syphilis may occur in late latent syphilis, especially in people living with HIV in which the progression of syphilis may be atypical.

## Author contributions

**LP**, contributed to the drafting and revising of the article. **LP**, **IR, DA**, **CC**, contributed to the treatment and management of the patient. **AC,** contributed to the conceptualization and critical revision of the original draft.

Author Agreement Statement.

We the undersigned declare that this manuscript is original, has not been published before and is not currently being considered for publication elsewhere.

We confirm that the manuscript has been read and approved by all named authors and that there are no other persons who satisfied the criteria for authorship but are not listed. We further confirm that the order of authors listed in the manuscript has been approved by all of us.

We understand that the Corresponding Author is the sole contact for the Editorial process. He/she is responsible for communicating with the other authors about progress, submissions of revisions and final approval of proofs Signed by all authors as follows:

## CRediT authorship contribution statement

**Antonio Cascio:** Supervision, Writing – review & editing. **Cinzia Calà:** Investigation. **Dalila Arena:** Investigation. **Irene Russotto:** Investigation. **Luca Pipitò:** Data curation, Investigation, Visualization, Writing – original draft, Writing – review & editing.

## Consent

Written informed consent was obtained from the patients for use of images for publication.

## Ethical approval

N/A.

## Funding

None.

## Declaration of Competing Interest

The authors declare that they have no known competing financial interests or personal relationships that could have appeared to influence the work reported in this paper.
